# microRNAs are differentially regulated between MDM2-positive and negative malignant pleural mesothelioma

**DOI:** 10.18632/oncotarget.7666

**Published:** 2016-02-24

**Authors:** Robert Fred Henry Walter, Claudia Vollbrecht, Robert Werner, Jeremias Wohlschlaeger, Daniel Christian Christoph, Kurt Werner Schmid, Fabian Dominik Mairinger

**Affiliations:** ^1^ Ruhrlandklinik, West German Lung Centre, University Hospital Essen, University of Duisburg-Essen, Essen, Germany; ^2^ Institute of Pathology, University Hospital Essen, University of Duisburg-Essen, Essen, Germany; ^3^ Institute of Pathology, University Hospital Cologne, Köln, Germany; ^4^ Institute of Pathology, Division of Molecular Pathology, Charité, Berlin, Germany; ^5^ Department of Medical Oncology, West German Cancer Centre, University Hospital Essen, University of Duisburg-Essen, Essen, Germany

**Keywords:** MDM2, microRNA, pleural mesothelioma, NanoString nCounter

## Abstract

**Background:**

Malignant pleural mesothelioma (MPM) is a highly aggressive tumour first-line treated with a combination of cisplatin and pemetrexed. MDM2 and P14/ARF (CDKN2A) are upstream regulators of TP53 and may contribute to its inactivation. In the present study, we now aimed to define the impact of miRNA expression on this mechanism.

**Material and Methods:**

24 formalin-fixed paraffin-embedded (FFPE) tumour specimens were used for miRNA expression analysis of the 800 most important miRNAs using the nCounter technique (NanoString). Significantly deregulated miRNAs were identified before a KEGG-pathway analysis was performed.

**Results:**

17 miRNAs regulating *TP53*, 18 miRNAs regulating *MDM2*, and 11 miRNAs directly regulating *CDKN2A* are significantly downregulated in MDM2-expressing mesotheliomas. TP53 is downregulated in MDM2-negative tumours through miRNAs with a miSVR prediction score of 11.67, RB1 with a prediction score of 8.02, MDM2 with a prediction score of 4.50 and CDKN2A with a prediction score of 1.27.

**Conclusion:**

MDM2 expression seems to impact miRNA expression levels in MPM. Especially, miRNAs involved in TP53-signaling are strongly decreased in MDM2-positive mesotheliomas. A better understanding of its tumour biology may open the chance for new therapeutic approaches and thereby augment patients' outcome.

## INTRODUCTION

Malignant pleural mesothelioma (MPM) is a biologically highly aggressive tumour arising from the pleura leading to a dismal prognosis [1–5].

Nowadays, cisplatin is the drug of choice for the treatment of MPM, and carboplatin seems to have comparable efficacy [1, 6–10]. Combined with antifolates they are considered as the most effective regimen for MPM [10–13]. However, patients only show response rates of approximately 40% with a progression-free survival (PFS) of 5.7 months [14].

In contrast to other solid tumours, mutations of the *TP53* gene are extremely rare in MPM, so other mechanisms such as deletion of the locus or methylation contribute to inactivation of TP53 [15–17]. Overexpression of MDM2 in some tumour types can lead to a loss of TP53 regulatory function in cancer cells by its increased proteasomal degradation [18–23]. P14/ARF, the physiological inhibitor of MDM2 is recognized as a tumour suppressor and induces cell cycle arrest in a TP53-dependent and -independent manner [24–27]. Analysis of the signalling relationship between these genes indicates an additional role of RB1 in this signalling network [28–30]. In previous studies, we have demonstrated that MDM2 is strongly expressed in the nuclei in approximately 25% of MPM affecting only epithelioid or the epithelioid components of biphasic MPM [23, 31]. Moreover, patients with MDM2-positive MPM showed a significantly decreased overall survival (OS) and progression-free survival (PFS) compared to MDM2-negative MPM [23, 31]. This might be explained by a significantly decreased or completely abolished TP53 activity and/or stability mediated by an overexpression of MDM2 [18–22]. A promising approach to explain the differences in MDM2 expression is a differential expression of miRNAs [32], targeting some of these key enzymes and thereby reducing expression levels of those.

The recent study is designed to analyse the miRNA profile of pleural mesotheliomas with respect to immunohistochemical MDM2 expression (score 0 versus score ≥1) using the nCounter system (NanoString), a hybridization-based digital detection method that can be used to analyse mRNA, miRNA and DNA [31, 33–36].

## RESULTS

136 miRNAs significantly differentially regulated between MDM2-positive and -negative samples were identified. 39 of them showed a p-value <0.01 and six of them (miR-106-5p, miR-15b-5p, miR24, miR-29a, miR-29c and miR-130a) a p-value <0.001.

Six miRNAs with p<0.01 were increased, whereas the remaining were decreased in MDM2-expression positive tumours.

All significant p-values are summarized in suppl. Table 1.

**Figure 1:**
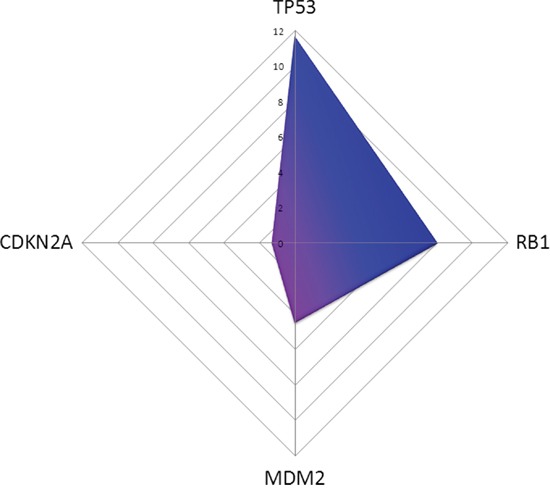
In silico prediction of miRNAs affecting TP53 pathway members in MDM2-negativ tumours compared to MDM2-positive ones is shown *TP53* is downregulated with a prediction score (PS) of 11.67, *RB1* with a PS of 8.02, *CDKN2A* with a PS 1.27 and *MDM2* with a PS of 4.50.

Furthermore, 17 miRNAs inhibiting *TP53*, 18 miRNAs binding *MDM2* and eleven miRNAs suppressing *CDKN2A* were significantly decreased in MDM2-expressing mesotheliomas. Of note, five miRNAs (miR-29a, miR-29b, miR-29c, miR-125a, miR-125b) binding all three targets, five regulating both *TP53* and *CDKN2A* (let-7a, let-7c, let-7d, let-7e, let-7g), and three binding both *MDM2* and *TP53* (miR-34a, miR-145, miR-185) were found to be significantly decreased, respectively. *TP53* is predicted to be suppressed in MDM2-negative tumours by miRNAs with a prediction score (PS) of 11.67, *RB1* with a PS of 8.02, *CDKN2A* with a PS 1.27 and *MDM2* with a PS of 4.50 (Figure 1).

A summary of the miRNAs, the affected target, correlating p-values and correlation coefficients is given in Table 1.

**Table 1:** An overview of significances of miRNAs directly regulating TP53-pathway members in MDM2-expressing and non-expressing tumours is shown

Target	miRNA Expression	p-Value	Correlation Coefficient
TP53/CDKN2A/MDM2	hsa-miR-125a	0.0078	−0.6030
TP53/CDKN2A/MDM2	hsa-miR-125b	0.0019	−0.6316
TP53/CDKN2A/MDM2	hsa-miR-29a	0.0000	−0.4055
TP53/CDKN2A/MDM2	hsa-miR-29b	0.0344	−0.4055
TP53/CDKN2A/MDM2	hsa-miR-29c	0.0000	−0.4055
TP53/CDKN2A	hsa-let-7a	0.0014	−0.5711
TP53/CDKN2A	hsa-let-7c	0.0117	−0.5711
TP53/CDKN2A	hsa-let-7d	0.0014	−0.5711
TP53/CDKN2A	hsa-let-7e	0.0142	−0.5711
TP53/CDKN2A	hsa-let-7g	0.0246	−0.5711
MDM2/TP53	hsa-miR-34a	0.0006	
MDM2/TP53	hsa-miR-145	0.0063	−0.3741
MDM2/TP53	hsa-miR-185	0.0470	−0.2059
CDKN2A	hsa-miR-340	0.0470	−0.1303
MDM2	hsa-miR-140	0.0063	−0.1286
MDM2	hsa-miR-223	0.0206	−0.2969
MDM2	hsa-miR-23b	0.0011	−0.1197
MDM2	hsa-miR-142	0.0246	
MDM2	hsa-miR-191	0.0008	
MDM2	hsa-miR-331	0.0403	
MDM2	hsa-miR-605	0.0292	
MDM2	hsa-miR-548d	0.0142	
MDM2	hsa-miR-374b	0.0172	−0.1462
MDM2	hsa-miR-383	0.0292	−0.1209
TP53	hsa-miR-19b	0.0031	−0.2945
TP53	hsa-miR-218	0.0050	−0.1678
TP53	hsa-miR-22	0.0078	−0.1955
TP53	hsa-miR-27b	0.0019	−0.5795

Some pathways are stimulated in MDM2-positive tumours by decreased miRNA expression. *In silico*, the most influenced KEGG-pathways between MDM2-positive and negative tumours with respect to the expression levels are Ribosome (eight targets, PS 36.24), Wnt signaling pathway (122 targets, PS 29.45), metabolism of xenobiotics by cytochrome P450 (seven targets, PS 24.14), MAPK signaling pathway (184 targets, PS 23.49), focal adhesion (145 targets, PS 21.53), regulation of actin cytoskeleton (152 targets, PS 19.46), axon guidance (99 targets, PS 19.26), colorectal cancer (73 targets, PS 18.11), oxidative phosphorylation (35 targets, PS 16.49) and TGF-beta signaling pathway (74 targets, PS 15.87).

**Figure 2:**
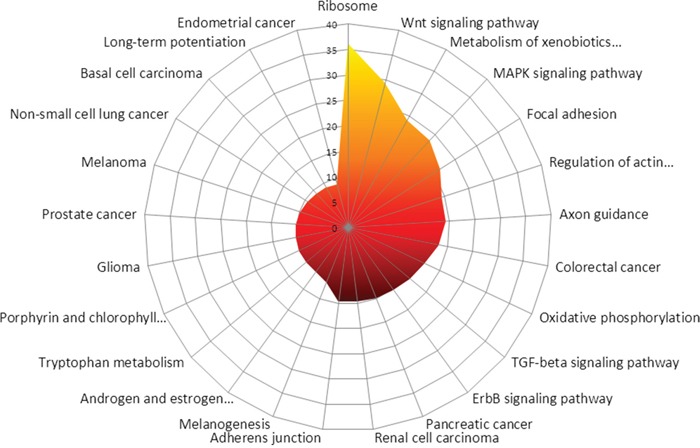
In silico prediction of the most affected KEGG-pathways between MDM2-positive and negative tumours is shown Most likely affected are Ribosome, Wnt signaling pathway, Metabolism of xenobiotics by cytochrome P450, MAPK signaling pathway, focal adhesion, regulation of actin cytoskeleton, Axon guidance, Colorectal cancer, Oxidative phosphorylation as well as TGF-beta signaling pathway.

An overview of all differentially stimulated pathways can be found in suppl. Table 2. The results are visualized in Figure 2.

## DISCUSSION

**Figure 3:**
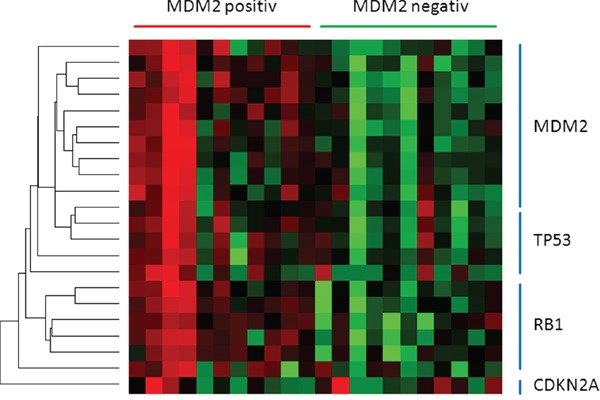
The heatmap presents miRNAs directing TP53 pathway members miRNAs regulating either *MDM2*, *TP53* or *RB1* are downregulated in MDM2-positiv tumours. *CDKN2A* seems to be unaffected.

Recently, a number of miRNAs targeting both *TP53* and *MDM2* have been identified. In *TP53* wild type plasmocytomas, TP53 can induce the expression of miR-192, miR-194 and miR-215, subsequently decreasing *MDM2* expression. Hypermethylation of the promoter region of all three miRNAs impairs the *MDM2* suppression, still resulting in a blunted TP53 function [38]. Additionally, miR-143, miR-45, miR-605, miR-34a and miR-29b are direct transcriptional targets of TP53 [44]. Together with the three former ones, they form a TP53-positive feedback loop by decreasing MDM2 and HDM4 expression levels [45, 46]. Nevertheless, 10 miRNAs suppressing *MDM2* showing low overall expression levels (Figure 3) but still seem to be associated to *TP53* activity and thereby are also significantly decreased in MDM2-positive tumours (Table 1).

*TP53* shows the strongest predicted suppression via miRNAs in MDM2-positive tumours with an overall PS of 11.67. Active TP53 stimulates the DROSHA complex, mediating the processing of tumour suppressor miRNAs [44, 47]. As already demonstrated, differences between epithelioid and sarcomatoid mesotheliomas regarding miR-34a expression were found [48]. These differences may be explained by the high incidence of loss of heterozygosity (LOH) in sarcomatoid mesotheliomas, whereas epitheloid subtypes more often show functional inhibition of TP53 via MDM2 [23, 31]. Furthermore, it has been reported that epigenetic silencing of miR-34 family members plays an important role in the pathogenesis of a majority of MPMs [49].

**Figure 4:**
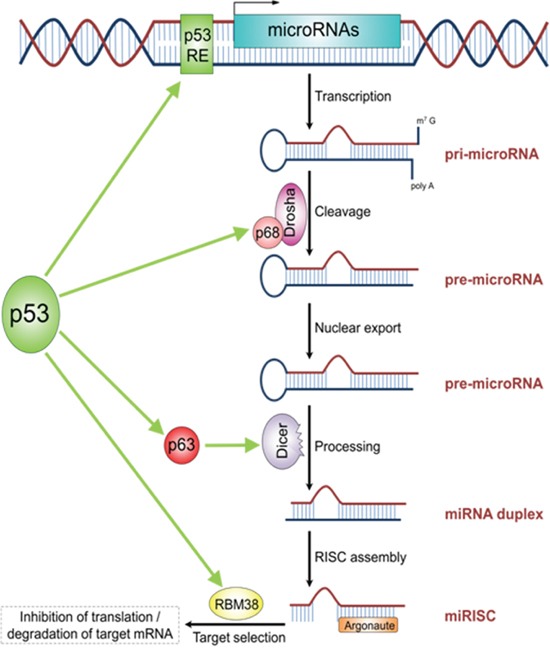
The schematic illustrations describes the role of TP53 in miRNA processing and regulation TP53 acts as transcription factor for some miRNAs. Additionally, TP53 plays a role in miRNA processing by directly interacting with P68 (DROSHA-complex), P63 (DICER-complex) as well as RBM38. The figure is adapted from Rokavec et al. Clin Exp Gastroenterol, 2014 [44].

MDM2 suppresses wild type TP53 leading to a more or less impaired but still specific transcriptional activity [50]. This may shift to other promoters e.g. controlling oncogenic miR-128b. Of note, activation of TP53 leads to an genome-wide change of miRNA pattern, indicating that TP53 has a complex role in regulating miRNAs (Figure 4) [51, 52].

Due to the high number of targets measured in a relatively low number of cases, a classical p-value adjustment by FDR or Bonferroni correction was not suitable. To our opinion, this study should be more seen like a screening study to get a first insight in possible regulatory mechanisms but therefore is still of importance to smooth the way for larger validation cohorts.

An interesting finding was the strong difference in activation of the ribosomal pathway predicted *in silico*. RPS6 encoding for the ribosomal protein S6, but also other ribosomal components, rendered high PS (suppl. Table 2), suggesting it plays an important role in pleural tumour progression. Multiple studies reported RPS6 as a predictive marker in pleural mesotheliomas with platinum-based chemotherapy [53, 54], but these findings may be coincidental. More likely, the MDM2 driven knock-down of TP53 can predict the response to platinum-based regimes and the subsequent regulation of ribosomal components acts as an indirect parameter. RL26 can directly bind TP53 and therefore form a positive feedback loop with MDM2 [55, 56].

In summary, 136 miRNAs significantly differentially expressed in MDM2-positive and -negative tumours were identified. Furthermore, 17 miRNAs directly binding *TP53*, 18 miRNAs directly suppressing *MDM2*, and eleven miRNAs directly inhibiting *CDKN2A* are significantly decreased in MDM2-expressing mesotheliomas. *TP53* is predicted to be strongly suppressed by miRNA depending on expression pattern, whereas the impact on *MDM2* itself is just moderate and on *CDKN2A* is even weaker. The present data lead to the conclusion that MDM2 expression level noticeably impacts the overall miRNA expression and thereby cellular pathways (e.g. ribosomal translation) in MPM. This can lead to a better understanding of the biology of MPM and may open the chance for new therapeutic approaches and thereby augment patients' outcome.

## MATERIALS AND METHODS

### Study design

For this exploratory miRNA study, twenty-four formalin-fixed paraffin-embedded (FFPE) tumour specimens were screened with respect to their MDM2 immunoexpression (twelve MDM2-positive and twelve MDM2-negative MPM) as described previously [31]. Tumor classification is based on the *WHO classification of tumours* guidelines (2004) [37], TNM-staging is based on the *UICC classification of malignant tumours* [38]. Both were confirmed by two experienced pathologists (JWO, KWS). The study included only patients with MPM, treated at the West German Cancer Centre or the West German Lung Centre between 2006 and 2009. Clinicopathological data including age, gender, histology and stages are summarized in Table 1. Surveillance for this study was stopped on August 31, 2014. The retrospective study was approved by the Ethics Committee of the Medical Faculty of the University Duisburg-Essen (identifier: 14-5775-BO). The investigation conforms to the principles outlined in the declaration of Helsinki.

### RNA extraction and RNA integrity assessment

According to the manufacturer's recommendations, three to five paraffin sections with a thickness of 4 μm per sample were deparaffinised with xylene prior to total RNA extraction including small RNAs using the miRNeasy FFPE kit (Qiagen, Venlo, Netherlands). RNA concentrations were measured using a Qubit 2.0 fluorometer with the appertaining RNA broad-range assay (Thermo Fisher Scientific, WA, USA). RNA integrity was assessed using a Fragment Analyzer (Advanced Analytical Inc., Ames, IA, USA).

### NanoString CodeSet design and expression quantification

The commercially available human miRNA V2.1 code set containing probes and miRTags for the 800 most important miRNAs described in the context of cancerogenic events was chosen for miRNA expression analysis. Probe sets and miRTags for each target in the CodeSet were designed and synthesized by NanoString Technologies (Seattle, WA, USA). 200 ng total RNA of each sample was processed. For the sample preparation, the high-sensitivity program was chosen. The cartridge was read with maximum sensitivity (555 FOV).

### NanoString data processing and statistical analysis

All statistical analyses were calculated with the R statistical programming environment (v2.15.2). NanoString data processing was done using the NanoStringNorm package [34]. Considering the counts obtained for positive control probe sets raw NanoString counts for each gene were subjected to a technical factorial normalization. Mean background plus 2x standard deviations were subtracted for background correction. Additionally, samples with less than mean background plus 2x standard deviations were interpreted as not expressed to overcome basal noise. After this procedure a biological normalization using the reference genes *ACTB* and *GAPDH* included in the CodeSet was performed. For *in silico* prediction of the functional impact of miRNAs we used DIANA-microT v4.0 [39], PicTar 4-way [40] as well as TargetScan5 [41]. KEGG-Pathway analysis was performed by the DIANA-mirPath tool for multiple miRNA analysis [42]. Analysis of already validated miRNA-target interactions was performed using the miRWalk database [43].

For statistical analysis of dichotomous factors such as gender and MDM2-positivity, the Wilcoxon Mann-Whitney rank sum test was applied. Associations between gene expression of tested genes and associations between gene expression and TNM-criteria were analysed by using the Spearman's rank correlation test.

The level of statistical significance was defined as p≤0.05.

## SUPPLEMENTARY TABLES




