# Supplementary material for: microRNAs are differentially regulated between MDM2-positive and negative malignant pleural mesothelioma
Source: Oncotarget. 2016 Feb 24;7(14):18713–21. doi: 10.18632/oncotarget.7666 (PMC4951323; doi:10.18632/oncotarget.7666)
Supplement: Supplementary file 1 [file oncotarget-07-18713-s001.pdf]

## SUPPLEMENTARY TABLES

**Supplementary Table 1: Overview of all significant miRNAs including the calculated p-values**

| miRNA Expression | p-Value | miRNA Expression | p-Value | miRNA Expression | p-Value |
|---|---|---|---|---|---|
| miR-106-5p | 0.000 | miR-105-5p | 0.014 | miR-1908 | 0.029 |
| miR-15b-5p | 0.000 | miR-1225-5p | 0.014 | miR-301b | 0.029 |
| miR-24-3p | 0.000 | miR-1275 | 0.014 | miR-376a-3p | 0.029 |
| miR-29a-3p | 0.000 | miR-1307-3p | 0.014 | miR-383 | 0.029 |
| miR-29c-3p | 0.000 | miR-139-5p | 0.014 | miR-412 | 0.029 |
| miR-130a-3p | 0.000 | miR-147b | 0.014 | miR-4425 | 0.029 |
| miR-34a-5p | 0.001 | miR-25-3p | 0.014 | miR-4458 | 0.029 |
| miR-191-5p | 0.001 | miR-3180-5p | 0.014 | miR-510 | 0.029 |
| miR-301a-3p | 0.001 | miR-320c | 0.014 | miR-518b | 0.029 |
| miR-93-5p | 0.001 | miR-320d | 0.014 | miR-545-3p | 0.029 |
| let-7i-5p | 0.001 | miR-361-3p | 0.014 | miR-605 | 0.029 |
| miR-23b-3p | 0.001 | miR-378a-3p-miR-378i | 0.014 | miR-769-3p | 0.029 |
| miR-497-5p | 0.001 | miR-431-5p | 0.014 | miR-10a-5p | 0.034 |
| let-7d-5p | 0.001 | miR-548d-3p | 0.014 | miR-1178 | 0.034 |
| let-7a-5p | 0.001 | miR-548w | 0.014 | miR-1206 | 0.034 |
| miR-125b-5p | 0.002 | miR-559 | 0.014 | miR-1263 | 0.034 |
| miR-27b-3p | 0.002 | miR-610 | 0.014 | miR-148a-3p | 0.034 |
| miR-107 | 0.003 | miR-651 | 0.014 | miR-202-3p | 0.034 |
| miR-1197 | 0.003 | miR-660-5p | 0.014 | miR-2114-5p | 0.034 |
| miR-19b-3p | 0.003 | miR-885-3p | 0.014 | miR-29b-3p | 0.034 |
| miR-30e-5p | 0.004 | miR-95 | 0.014 | miR-3168 | 0.034 |
| miR-106b-5p | 0.005 | miR-1234 | 0.017 | miR-3184-5p | 0.034 |
| miR-1260a | 0.005 | miR-199a-5p | 0.017 | miR-3187-3p | 0.034 |
| miR-1266 | 0.005 | miR-199b-5p | 0.017 | miR-320b | 0.034 |
| miR-1299 | 0.005 | miR-26b-5p | 0.017 | miR-371a-3p | 0.034 |
| miR-218-5p | 0.005 | miR-324-5p | 0.017 | miR-624-3p | 0.034 |
| miR-361-5p | 0.005 | miR-374b-5p | 0.017 | miR-628-3p | 0.034 |
| miR-140-5p | 0.006 | miR-4531 | 0.017 | miR-633 | 0.034 |
| miR-145-5p | 0.006 | miR-127-3p | 0.021 | miR-654-5p | 0.034 |
| miR-148b-3p | 0.006 | miR-132-3p | 0.021 | miR-665 | 0.034 |
| miR-3196 | 0.006 | miR-20a-5p-miR-20b-5p | 0.021 | miR-744-5p | 0.034 |

(*Continued*)

| miRNA Expression | p-Value | miRNA Expression | p-Value | miRNA Expression | p-Value |
|---|---|---|---|---|---|
| miR-4741 | 0.006 | miR-2116-5p | 0.021 | miR-933 | 0.034 |
| miR-941 | 0.006 | miR-223-3p | 0.021 | miR-1283 | 0.040 |
| miR-125a-5p | 0.008 | miR-30d-5p | 0.021 | miR-302a-3p | 0.040 |
| miR-199a-3p-miR-199b-3p | 0.008 | let-7g-5p | 0.025 | miR-331-3p | 0.040 |
| miR-22-3p | 0.008 | miR-1250 | 0.025 | miR-4454 | 0.040 |
| miR-342-3p | 0.008 | miR-142-3p | 0.025 | miR-518c-3p | 0.040 |
| miR-374a-5p | 0.008 | miR-3180 | 0.025 | miR-658 | 0.040 |
| miR-99a-5p | 0.008 | miR-32-5p | 0.025 | miR-185-5p | 0.047 |
| miR-99b-5p | 0.008 | miR-4286 | 0.025 | miR-340-5p | 0.047 |
| miR-15a-5p | 0.010 | miR-450b-3p | 0.025 | miR-507 | 0.047 |
| let-7c | 0.012 | miR-564 | 0.025 | miR-509-3p | 0.047 |
| miR-151a-3p | 0.012 | miR-720 | 0.025 | miR-548ag | 0.047 |
| miR-195-5p | 0.012 | miR-1226-3p | 0.029 | miR-764 | 0.047 |
| let-7e-5p | 0.014 | miR-1231 | 0.029 | | |
| miR-100-5p | 0.014 | miR-152 | 0.029 | | |

**Supplementary Table 2: Overview of all calculated KEGG-Pathway stimulation effects predicted by the DIANA-mirPath tool for multiple miRNA analysis.** The exact KEGG-paythway name, the ID, the number of affected targets within this pathway as well as the prediction score and the Pathway names are provided.

See Supplementary File S1
